# Spliceosomal repression: unleashing human cell totipotency

**DOI:** 10.1038/s41392-024-01966-2

**Published:** 2024-09-23

**Authors:** Felipe F. Lüttmann, Kee-Pyo Kim, Johnny Kim

**Affiliations:** 1 The Center for Cardiovascular Regeneration and Immunology at TRON - Translational Oncology at the University Medical Center of the Johannes Gutenberg-University Mainz gGmbH, Mainz, Germany; 2 Department of Medical Life Sciences, College of Medicine, The Catholic University of Korea, Seoul, Republic of Korea; 3 Vascular Biology and Therapeutics Program and Department of Comparative Medicine, Yale University School of Medicine, New Haven, CT USA

**Keywords:** Totipotent stem cells, Biological models

In a recent publication in *Cell*, Li et al. reported that inhibiting the spliceosome in human pluripotent stem cells (hPSCs) can induce a stable totipotent state.^1^ This study signifies the importance of post-transcriptional regulation in controlling developmental potential and provides a powerful model system for studying the process of human pre-implantation development in vitro.

**Fig. 1:**
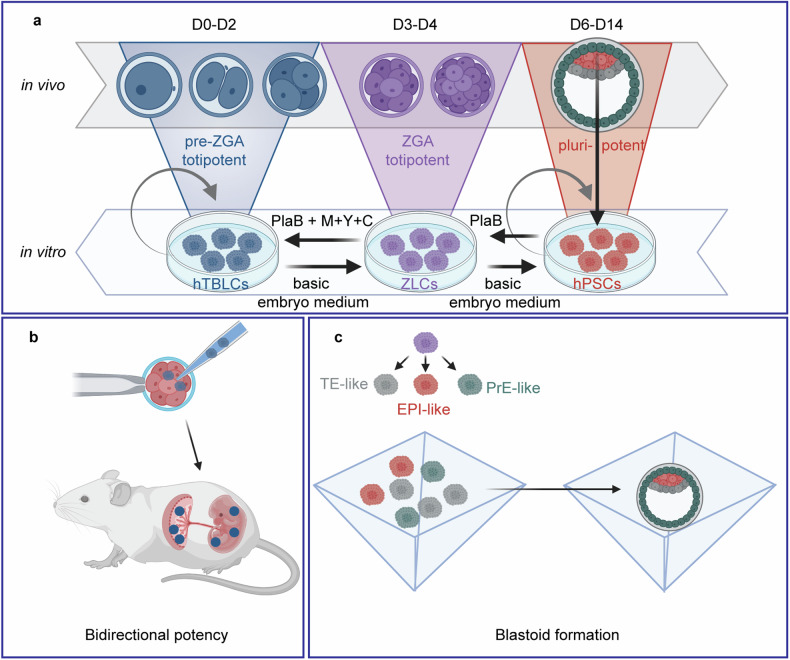
Human totipotent blastomere-like cells (hTBLCs) as a model for pre-implantation development. **a** Pluripotent hPSCs can be derived from the epiblast of human embryos. Upon treatment with the spliceosomal inhibitor pladienolide B (PlaB), hPSCs reprogram to meta-stable ZGA-like cells (ZLCs) that transcriptionally resemble totipotent embryos when zygotic genome activation (ZGA) occurs. By the addition of minocycline, Y27632, and CHIR99021 (+M+Y+C), ZLCs can be further reprogrammed to stable pre-ZGA-like hTBLCs. **b**, **c** hTBLCs can contribute to embryonic and extra-embryonic tissues in chimeric embryos (**b**) and can re-differentiate into ZLCs and then into trophectoderm (TE)-like, epiblast (EPI)-like and primitive endoderm (PrE)-like cells which can self-organize into blastocyst-like structures (blastoids) (**c**). Generated with BioRender.com

Totipotency refers to a cell’s ability to differentiate into all embryonic and extra-embryonic tissues, allowing it to form a complete organism. This totipotent potential exists only in the earliest stage of embryonic development, lasting for four days after fertilization in humans. During this period, at the 8-cell stage, the embryo starts to express genes from its genome for the very first time, a process coined zygotic genome activation (ZGA). Investigating ZGA is challenging because embryos only consist of few cells at this time and access to human material is further restricted by ethical issues. Thus, a longstanding goal has been to identify ways to achieve stable culture of totipotent stem cells (Fig. 1).

Along these lines, recently it was reported that a defined inhibitor cocktail against MEK, tankyrase, EZH2, and histone deacetylase class I in combination with leukemia inhibitory factor (LIF) in hPSCs can produce so-called 8-cell-like cells (8CLCs), which mimic epigenetic and transcriptional features of human embryos during ZGA.^2^ 8CLCs exhibit totipotent potential since they can contribute to extra-embryonic tissues. However, three major constraints have remained: (i) 8CLCs can only be cultured for a few passages before reverting back to a pluripotent state, (ii) 8CLCs still express pluripotent marker genes, indicating incomplete silencing of the pluripotent network, and (iii) embryonic stages preceding the 8-cell stage and ZGA are not naturally replicated in 8-cell-like cells (8CLCs). Therefore, exploring embryonic stages before ZGA is not feasible within this experimental system.

ZGA results in a transcriptional burst of thousands of genes, many of which are only functionally relevant much later in development. Interestingly, such developmental genes tend to possess more and longer introns than genes that encode regulators of the totipotent stage.^3^ Thus the lack of a functional splicing machinery at the totipotent stage might facilitate protecting the embryo against harmful misexpression of developmental genes at the protein level.^3^ This hypothesis is supported by the observations of Li et al., which show that treatment with the splicing inhibitor pladienolide B (PlaB) can reprogram hPSCs to ZGA-like cells (ZLCs) in a dosage- and time-dependent manner, as revealed by transcriptional profiling. Remarkably, treating mouse embryonic stem cells with the splicing inhibitor pladienolide B (PlaB) is sufficient to reprogram them to a totipotent state, which supports this hypothesis. In their current work, Li et al. now tested if spliceosomal inhibition exerts similar effects in humans. Indeed, treatment with PlaB reprogrammed hPSCs into ZGA-like cells (ZLCs) in a dosage- and time-dependent manner, as revealed by transcriptional profiling. Single-cell RNA-sequencing (sc-RNA-seq) showed that ZLCs resembled human embryonic day 3 embryos, while 8CLCs clustered with human embryonic day 4 embryos. This was further reflected by the fact that some ZGA-associated genes were expressed by both ZLCs and 8CLCs, while others were specific to either cell type. Intriguingly, ZLCs did not express genes from the *DUX*- and *TPRX*-families which have been previously shown to be involved in the regulation of ZGA in humans and mice.^4,5^ Unlike 8CLCs, ZLCs notably lacked expression of pluripotent marker genes, which exhibited intron retention and eventual silencing after treatment with PlaB. However, similar to 8CLCs, ZLCs could not be stably cultured. To overcome this, Li et al., supplemented the PlaB-containing medium with three additional compounds: minocycline, Y27632, CHIR99021, which are chemical inhibitors of PARP1, ROCK, and GSK3, respectively. Remarkably, this cocktail coined MYCP, enabled stable culture of PlaB-treated cells with high viability, proliferation rates, and chromosomal stability. As shown by RNA-seq, this cocktail reprogrammed hPSCs in ZLCs after three to four passages and eventually into so-called human totipotent blastomere-like cells (hTBLCs) after eight passages. In contrast to 8CLCs and ZLCs, hTBLCs did not express pluripotency- or ZGA-associated genes but instead resembled a pre-ZGA state similar to the zygote, 2- or 4-cell stage embryos.

The cogency of the hTBLC model was further demonstrated by the ability of hTBLCs to differentiate after withdrawal of the MYCP inhibitor cocktail. Four hours after the medium exchange, pre-ZGA genes became downregulated while ZGA genes were upregulated, partially mimicking the activation of the human embryonic genome. Notably, however, the ZLC-like transcriptome did not include genes from the *DUX*- and *TPRX*-families or their direct targets. After 24–72 h of differentiation, sc-RNA-seq analysis revealed lineage segregation into trophectoderm-like, primitive endoderm-like, and epiblast-like cells. Remarkably, upon withdrawal of inhibitors, hTBLC-derived cells self-organized into blastocyst-like structures (blastoids) without any additional external signaling manipulation. This was confirmed through morphological assessment, immunofluorescence for lineage markers, and sc-RNA-seq. hTBLCs were eventually able to differentiate into the three germ layers (endo-, ecto- and mesoderm) in teratoma and embryonic body formation assays. Finally, the developmental potential of hTBLCs was assessed in vivo by their injection into 8-cell-stage mouse embryos and their subsequent transplantation into pseudopregnant mice. Intriguingly, the totipotent hTBLC-derived cells displayed bidirectional developmental potential as they were able to contribute to the entire developing conceptus, including the extra-embryonic chimeric placenta.

In conclusion, hTBLCs are the first reported in vitro approach modeling the earliest phase of human development. These stable totipotent stem cells offer significant potential for advancing our understanding of human development. However, several questions remain to be addressed to validate this model and to understand to which degree it reflects human development in vivo: (i) How does the inhibition of the spliceosomal machinery and subsequent downregulation of the pluripotent network result in resurrection of totipotency without causing cell death or differentiation? (ii) Do hTBLC-derived differentiating cells display genomic imprinting and X-chromosomal inactivation following the temporal and spatial dynamics of their in vivo counterparts? (iii) How can hTBLCs, reflecting a pre-ZGA state, be stably cultured, while 8CLCs or ZLCs, resembling a ZGA-like state, cannot be propagated over several passages? (iv) TPRX- and DUX- family members have been identified as essential regulators of ZGA in humans and mice, respectively. Interestingly, hTBLC-derived ZLC-like intermediate states do not express these transcription factors or their targets. This raises questions whether hTBLC-derived cells can replicate the full spectrum of transcriptional changes during ZGA and the transition from totipotency to pluripotency.
